# Concomitant Tuberculosis and Carcinoma Colon: Coincidence or Causal Nexus?

**DOI:** 10.4103/1319-3767.70619

**Published:** 2010-10

**Authors:** Saurav Chakravartty, Gautam Chattopadhyay, Dipankar Ray, Chandan Roy Choudhury, Subhayan Mandal

**Affiliations:** Department of Surgical Gastroenterology, Medical College, Kolkata, India; 1 Department of General Surgery, Medical College, Kolkata, India

**Keywords:** Carcinoma colon, coexisting TB and carcinoma, etiological relationship, intestinal tuberculosis

## Abstract

Two rare cases of adenocarcinoma of the caecum and ascending colon concomitant with tuberculosis at the same site are reported. The plausibility of an aetiological relationship between the two pathological conditions has been discussed along with a review of the relevant literature. Tuberculosis complicating malignant disease is a diagnostic and therapeutic challenge; and the likelihood of the two occurring together should be kept in mind especially in tuberculosis endemic areas and in patients with equivocal symptoms.

The coexistence of carcinoma and tuberculosis is unusual and their association has bewildered surgeons and scientists alike for over two centuries. World literature reports only 67 cases of tuberculosis (TB) and colonic carcinoma occurring simultaneously.[1–3] A few examples of the same in the Indian context are on record but adenocarcinoma and tuberculosis occurring at the same site is exceedingly rare. This prompted us to report these two cases and review the possibility of an etiological relationship between the two pathological conditions.

## CASE REPORTS

### Case 1

In the first case, a 65-year-old female presented with complaints of low-grade fever and abdominal pain for 3 days and history of similar intermittent episodes for the preceding 6 months. The pain was dull, aching, and continuous in character. The patient also reported fatigue and weight-loss over the past 6 months. Examination revealed a 5 cm × 3 cm well-defined firm lump in the right iliac fossa. Hematological investigations showed normocytic normochromic anemia with a raised ESR. Chest roentgenogram was normal. A Mantoux test resulted in an induration of 15 mm. Polymerase chain reaction of blood for *Mycobacterium tuberculosis* showed a positive result. A diagnosis of ileocaecal tuberculosis was made and antitubercular therapy was instituted. However, the lump did not resolve and the patient returned with development of frank obstructive symptoms. Laparotomy was undertaken. A large mass involving the terminal ileum, caecum, and part of the ascending colon with multiple lymph nodes along the draining vessels was discovered. A limited colectomy was performed with diagnosis of tuberculosis. The postoperative period was uneventful.

Histopathology revealed moderately differentiated mucinous infiltrating adenocarcinoma. Epithelioid granulomas with Langhans and foreign body giant cells were present. The lymph nodes also showed epithelioid granulomas with Langhans and foreign body giant cells, two among them showing caseation. None revealed metastatic carcinomatous deposit. A diagnosis of moderately differentiated mucin-secreting infiltrating Duke’s stage A adenocarcinoma of the ileo-caecum and ascending colon associated with tuberculosis was made.

The patient completed the full course of anti-tubercular drugs and was followed up with serial CEA levels, which remained within normal limits.

### Case 2

**Figure 1:**
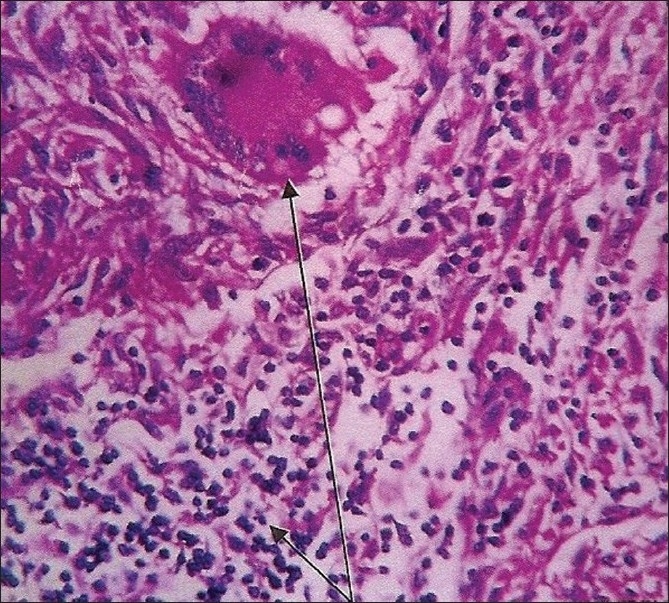
Grade I adenocarcinoma coexisting with tuberculosis

The second case was a 45-year-old man who presented with iron deficiency anemia and hepato-splenomegaly but no bowel irregularities or symptoms suggestive of tuberculosis. He underwent an extensive hematological evaluation but no abnormality was found. Five months later he developed a lump in the right iliac fossa. A colonoscopy revealed a concentrically thickened wall of the ascending colon and a biopsy confirmed an adenocarcinoma. He underwent a right hemicolectomy. A large cauliflower growth (7 cm) was found in the caecum. Histopathology revealed a well-differentiated adenocarcinoma with subserosal invasion but no angiovascular involvement. Surprisingly, there was evidence of granulomatous inflammation with caseous necrosis, epithelioid cell granuloma, and Langhans-type giant cells [Figure 1]. Similar granuloma, but without metastasis, was seen in the lymph nodes. The margins of the specimen were free of tumor. The patient was on anti-tubercular medication, and on follow up and on serial CEA examinations he was found to be doing well.

## DISCUSSION

Arguably, the first published description of coexisting tuberculosis and carcinoma was that of Boyle who described “cavitation cancereuse” as one of the six types of tuberculosis.[4] The association of tuberculosis and cancer has since been recorded in most organs by various authors. Carcinoma in different parts of the colon with intestinal tuberculosis have been reported by Paustian.[5] Kaplan *et al*. found TB complicating neoplastic disease in only 4 out of 6472 patients with carcinoma of the colon, a prevalence of 6/10,000.[6] Indian researchers have found a higher frequency of coexistent disease.[7]

Some Indian authors have proposed that the association of carcinoma and tuberculosis is coincidental; the argument being that compared to the high incidence of abdominal tuberculosis in India, the cases of coexisting tuberculosis and carcinoma are very few.[7] This may be true in some cases particularly when the neoplasm originates at a site distant from the tubercular focus. However, to put the simultaneous occurrence of the two conditions at the same site down to mere coincidence is far too simplistic.

Some diseases like ulcerative colitis, Crohns disease, and schistosomiasis predispose to malignancy. Chronic inflammatory mucosal damage initiating a sequence of metaplasia and dysplasia results in neoplastic change. Evidence also suggests that pulmonary scarring of tuberculous etiology play a role in the generation of some lung cancers, usually adenocarcinomas originating in the peripheral portion of the lung. Drawing parallels it may be postulated that the ulcerative lesions of intestinal tuberculosis are precursors of carcinomas and this possibility was suggested by Japanese researchers.[8] These carcinomas arose as a result of repeated insults by way of erosions, ulceration, and consequent regeneration.

On the other hand, it is also universally accepted that factors that disturb host immunity increase susceptibility to active tubercular infection, either exogenously or endogenously. Severe weight loss or malnutrition related to an advanced neoplastic disease is such a factor. Conceivably invasion of a dormant tubercular lesion by carcinoma could lead to activation and endogenous reinfection. Locally produced tumor peptides or antigens may also upset the milieu of a granuloma and allow the TB organisms to proliferate.[6] We are inclined to believe that this is true in our case given the age of the patient and the lack of a previous history of active tuberculosis. The other question is whether this patient needed to have a reoperation to complete the right hemicolectomy oncologically.

There have not been any reports of patients presenting with anemia with an underlying colonic carcinoma with coexisting tuberculosis. So, the second case highlights the need for a complete evaluation of patients with unaccounted iron deficiency anemia. This should include an upper gastrointestinal endoscopy and colonoscopy even in the absence of obvious gastro-intestinal symptoms.

The best treatment in these cases is difficult to decide as pre-operative diagnosis of such a coexisting dual pathology is virtually impossible.[9]

## CONCLUSION

The primary pathology is a matter of conjecture. Since a preoperative diagnosis in such cases is usually not possible, a case of right iliac fossa lump with evidence of tuberculosis should also be treated with a suspicion of co-existing malignancy especially in patients who fail to respond to anti-tubercular drugs.
